# Prediction of early postoperative recurrence of hepatocellular carcinoma by habitat analysis based on different sequence of contrast-enhanced CT

**DOI:** 10.3389/fonc.2024.1522501

**Published:** 2025-01-03

**Authors:** Yubo Zhang, Hongyan Ma, Peng Lei, Zhiyuan Li, Zhao Yan, Xinqing Wang

**Affiliations:** ^1^ Department of Hepatobiliary Surgery, General Hospital of Ningxia Medical University, Yinchuan, China; ^2^ School of Clinical Medicine, General Hospital of Ningxia Medical University, Yinchuan, China

**Keywords:** computed tomography (CT), early recurrence, habitat analysis, hepatocellular carcinoma, machine learning

## Abstract

**Aim:**

To develop a habitat imaging method for preoperative prediction of early postoperative recurrence of hepatocellular carcinoma.

**Methods:**

A retrospective cohort study was conducted to collect data on 344 patients who underwent liver resection for HCC. The internal subregion of the tumor was objectively delineated and the clinical features were also analyzed to construct clinical models. Radiomics feature extraction was performed on tumor subregions of arterial and portal venous phase images. Machine learning classification models were constructed as a fusion model combining the three different models, and the models were assessed.

**Results:**

A comprehensive retrospective analysis was conducted on a cohort of 344 patients who underwent hepatic cancer resection at one of the two centers. it was found that the combined SVM model yielded superior results after comparing various metrics, such as the AUC, accuracy, sensitivity, specificity, and DCA.

**Conclusions:**

Habitat analysis of sequential CT images can delineate distinct subregions within a tumor, offering valuable insights for early prediction of postoperative HCC recurrence.

## Introduction

The incidence of hepatocellular carcinoma (HCC) ranks sixth among malignant tumors globally, and it is the fourth leading cause of cancer-related mortality (1). The preferred treatment for HCC is surgical resection; however, research has indicated that approximately 70% of patients who undergo surgical resection experience recurrence within a span of 5 years (2–4). A postoperative time point of 2 years is used as the threshold to identify HCC recurrence as early and late recurrence. Various studies suggest that patients with early recurrence exhibit a significantly poorer prognosis than those with late recurrence (5). Consequently, accurate assessment of recurrence risk holds paramount importance in clinical decision-making and guiding personalized treatment strategies (6).

In recent years, radiomics has emerged as a promising non-invasive, high-throughput imaging technique for the diagnosis, treatment, and prognosis of tumors (7). Conventional radiomics studies typically analyze tumors as a whole, assuming their heterogeneity but well-mixed. However, solid cancers have significant spatial and temporal heterogeneity (8). They possess varying compositions and spatial distributions of cell populations within the same tumor (9). However, previous studies have overlooked local phenotypic variations within tumors (10). The technique of habitat analysis, which is a developing imaging analysis method, involves partitioning groups of voxels with similar tumor biology into subregions for enhanced visualization and quantification of intra-tumor heterogeneity. This approach has significant potential for personalized analysis (9, 11).

The use of hepatic contrast agent-enhanced MRI (CE-MRI) for habitat imaging reportedly has predictive value in determining microvascular invasion and recurrence-free survival after HCC resection (12, 13). The accessibility and cost-effectiveness of CT make it a preferred choice over MRI in clinical settings. However, there is currently a lack of multi-institutional studies that explore the extraction of subregion features from different sequences in CT for habitat analysis to predict early recurrence of HCC. This study was aimed at quantitatively and visually analyzing distinct subregions within the tumor based on habitat analysis of sequential CT images. We aimed to develop and validate an efficient and non-invasive preoperative model for predicting early postoperative recurrence of HCC, thus offering novel approaches and concepts for personalized treatment and clinical management of patients with HCC.

## Materials and methods

### Study population

**Figure 1:**
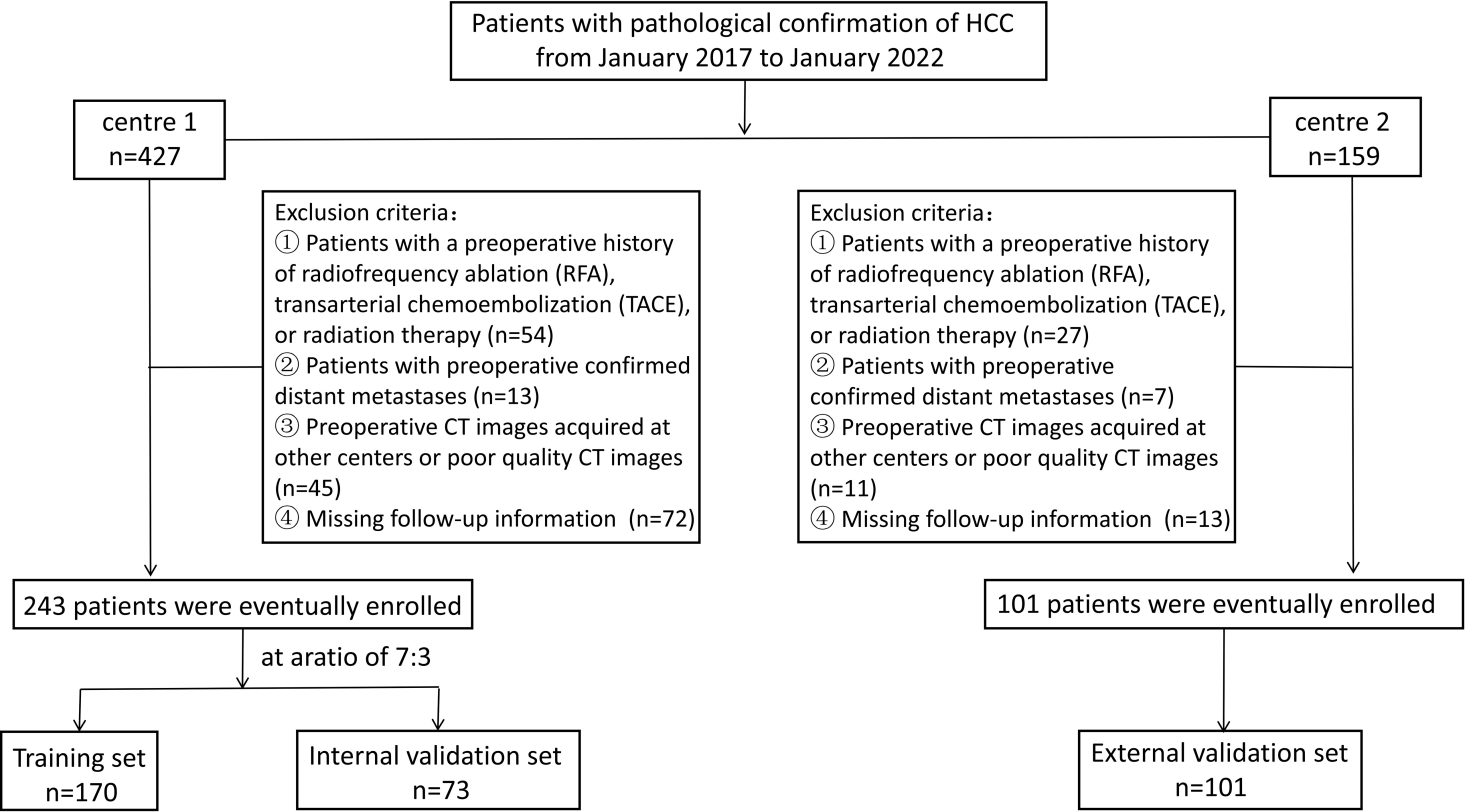
Patient selection flowchart.

This retrospective study was approved by the ethics committees of the participating institutions, and the need for patients’ written informed consent was waived because of the retrospective design. General Hospital of Ningxia Medical University (Ethics Approval Number: KYLL-2023-0232), People’s Hospital of Ningxia Hui Autonomous Region (Ethics Approval Number: 2023-LL-057). Patients who underwent hepatic resection for HCC from January 2017 to January 2022 at either of the two medical centers were included. The inclusion criteria were as follows: patients whose initial treatment plan was hepatectomy for HCC and postoperative pathology confirmed HCC and patients who underwent CT examination within one month before the operation. The exclusion criteria were as follows: (1) Patients with a preoperative history of undergoing radiofrequency ablation (RFA), transarterial chemoembolization (TACE), or radiation therapy; (2) patients with preoperatively confirmed distant metastases; (3) patients with preoperative CT images acquired at other centers or patients having poor-quality CT images. In addition, (4) patients with missing follow-up information. Patients recruited from center 1 were randomly assigned to training and internal validation datasets in a 7:3 ratio. Patients from center 2 comprised the external validation dataset. Patient selection flowchart is shown in **Figure 1**.

**Figure 2:**
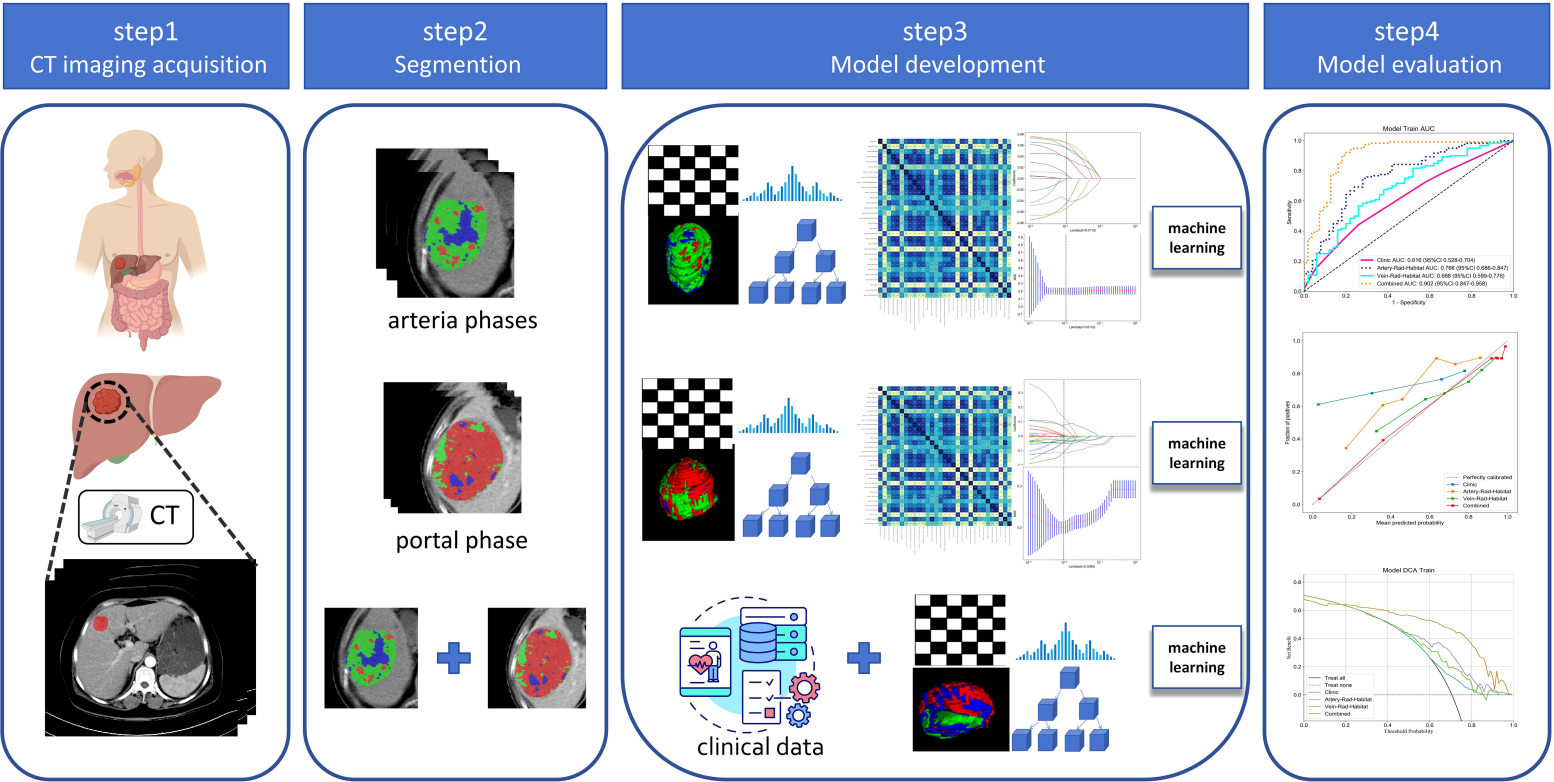
Flowchart of this study.

The medical records provided preoperative clinical data for the following parameters: age, sex, hypertension, diabetes, cirrhosis, hepatic virus infection, total bilirubin (Tbil), γ-glutamyl transferase (GGT), systemic immune-inflammation index (SII), lymphocyte-to-monocyte ratio (LMR), neutrophil-to-lymphocyte ratio (NLR), aspartate aminotransferase-to-lymphocyte ratio (ALR), platelet to lymphocyte ratio (PLR), alpha-fetoprotein (AFP), albumin (ALB), platelet count (PLT), aspartate transaminase (AST), alanine aminotransferase (ALT), maximum tumor diameter, and number of tumors. Patients were postoperatively followed up on an outpatient basis, with the follow-up commencing one month after discharge and scheduled every three months during the first year. The follow-up included serology (liver function tests and serum alpha-fetoprotein levels) and imaging (chest CT scan *and* abdominal CT or MRI). Second year onward, follow-up visits were scheduled every six months. The follow-up period extended until February 26, 2024, or until tumor recurrence, patient loss, or death. Early recurrence of HCC was defined as the emergence of new tumors within or outside the liver following treatment within 2 years postoperatively (14), with postoperative recurrence determined through imaging or pathological examinations. The time interval between hepatic resection and the first recurrence was defined as the time to recurrence (TTR) for HCC. The study flow is shown in **Figure 2**.

### CT acquisition and scanning parameters

For imaging, center 1 used a 256-slice spiral CT scanner (Brilliance iCT, Philips, Netherlands) or a 64-slice spiral CT scanner (SOMATOM Definition, SIEMENS, Germany), and center 2 used a 512-slice spiral CT scanner (Revolution Apex CT, GE, America) or a 256-slice spiral CT scanner (Brilliance iCT, Philips, Netherlands). For image acquisition, the patients were positioned in the supine position and underwent the scan in one breath-hold from the apex of the diaphragm to the lower edge of the pubic symphysis plane. Scanning parameters included a tube voltage of 100–120 kV and a tube current of 150–250 mA. The matrix size was set at 512 × 512 pixels. For the area of interest, layer thickness and spacing were both set at 1 mm. Contrast agent injection was performed using a double-barrel high-pressure syringe (Stellant, Medrad Company, United States). A non-ionic contrast agent (ioversol, concentration: 300 mgI/mL) was administered through the elbow vein at a total volume of 1.5–2 mL/kg with an injection rate of 2.5–3.0 mL/s. After the injection, saline flush with a volume of ~20 mL was performed before scanning during the arterial phase, which lasted for 30–35 s, followed by portal phase, which lasted for 60–65 s; delayed phase scanning was performed at 120–150 s.

### Tumor segmentation and habitat subregion generation

Tumor segmentation was performed using the ITK-SNAP software (version 3.6, www.itk-snap.org). Two experienced abdominal radiologists with 10 and 15 years of expertise independently evaluated all CT images. They conducted a blind assessment without access to clinical or pathological information and manually delineated the region of interest by precisely outlining the boundaries of the target lesion layer by layer. Before generating subregions, accurately localizing the tumor in the CT image is crucial. Each CT image was paired with a corresponding tumor mask with an identical shape, from which the tumor region was extracted. Subsequently, radiomics information within the tumor was extracted by considering each pixel within it as a center point and expanding it outward by 2 pixels in each direction, resulting in a sliding window of size 5 × 5 × 5; radiomics features within this window were then computed using Pyradiomics package (version: 2.12; https://pyradiomics.readthedocs.io/en/2.1.2/). Finally, all features were scaled to a range of 0 to 1 for subsequent clustering analyses.

Although increasing the window size and extracting more radiomics features can enhance noise immunity, the computational effort will exponentially increase with each additional pixel or radiomics feature in the window because of the requirement of performing feature extraction operations for every tumor pixel. Therefore, this study set the number of radiomics features to five, all of which were derived from Gray-Level Co-occurrence Matrix (GLCM), including contrast, difference entropy, joint energy, joint entropy, and correlation. GLCM can capture subtle texture variations in response to irregularities and complexity in images, making it a valuable tool for investigating tumor image heterogeneity (15). Ultimately, each pixel’s local radiomics information is transformed into a five-dimensional feature vector.

Pixels showing similar radiomics features indicate tissue homogeneity. We used a Gaussian mixture model clustering technique to identify comparable subregions within the tumor. The number of clusters, denoted as k, governs the granularity of the clusters. In this study, we set k to 3 based on previous reports that habitat area calculations with k = 3 yield greater robustness (16, 17). After clustering the internal regions of the tumor and assigning distinct color labels to each cluster, a cluster label map was generated to depict the overall distribution pattern.

### Radiomics feature extraction

Radiomics feature extraction was performed on tumor subregions of arterial and portal venous phase images. Preprocessing of CT images before feature extraction was done as follows. To minimize differences caused by scanning equipment and protocols, all CT images were first resampled to a voxel size of 1 × 1 × 1 mm^3^. Then, voxel intensity values were discretized by using a consistent bin width of 25 HU to minimize image noise and standardize intensities, thus ensuring uniform intensity resolution in all tumor images. Eight filters were applied, including wavelet, Laplacian of Gaussian (LoG), gradient, local binary pattern 3D, exponential, square, square root, and logarithm. Unfiltered (raw image) and filtered features were extracted for analysis using the open-source Python package PyRadiomics (version 3.0.1; https://pyradiomics.readthedocs.io/en/latest/index.html). For each region, a total of 1561 features were extracted and classified according to the following feature classes: 306 first-order features, 14 shape features, 374 GLCM features, 272 gray-level size zone matrix features, 272 gray-level run length matrix features, 82 neighboring gray tone difference matrix features, and 238 gray-level dependence matrix features. A total of 4683 radiomics features were extracted from the three habitat subregions of each sequence. PyRadiomics adheres to the image biomarker standardization initiative. Z-Score normalization was performed on the features extracted from the training set, internal validation set, and external validation set.

### Feature selection and model construction

To identify radiomics features with good reproducibility and low redundancy, the features were initially subjected to an independent-sample t-test to eliminate those with a *P* value exceeding 0.05. Then, for features showing high repeatability, Pearson correlation coefficients were computed to quantify their relationship with each other. Only the pairs of features that showed a correlation coefficient of >0.9 were retained. Finally, using the least absolute shrinkage and selection operator (LASSO) algorithm, stable radiomics features were incorporated into the LASSO regression analysis by constructing a penalty function, λ, to shrink some regression coefficients to force some features to zero. Based on the minimum value criterion, 10-fold cross-validation was performed to determine the optimal λ value. Radiomics features with non-zero coefficients were screened according to the model corresponding to the best λ value. Thus, independent and stable radiomics features were obtained. All screening features were standardized using the Z-score method, and the mean and variance were calculated for each column of features. Each column of characteristics was converted to a standard normal distribution by subtracting the mean and dividing by the variance. Finally, based on the features and their corresponding coefficients screened by the LASSO regression algorithm, a bar chart of the feature coefficients was drawn to assess the degree of importance of each feature. After feature fusion and screening, machine learning classification models were constructed using the scikit-learn machine learning library. The machine learning classification models include logistic regression (LR), support vector machine (SVM), decision tree (DT), random forest (RF), extremely randomized trees (ExtraTree), extreme gradient boosting (XGBoost), and multilayer perceptual machine (MLP). To reduce overfitting, 5-fold cross-validation was performed to select the best parameters for the classification model under training. Subjects’ work characteristic curves (ROC) were plotted, and the area under the curve (AUC) was calculated.

A univariable LR analysis was performed to identify the risk factors associated with early recurrence of HCC after resection on the basis of clinical characteristics. Comparing the results of arterial phase habitat radiomics and portal phase habitat radiomics modeling, the optimal model outputs in the arterial and portal phases were selected as arterial phase habitat radiomics signature (artery-rad-hab-sign) and portal phase habitat radiomics signature (portal-rad-hab-sign), respectively. Combining clinically relevant risk factors and habitat radiomics signatures, we employed a combined model. To comprehend and interpret the decision rules acquired by the combined machine learning model, SHapley Additive exPlanations (SHAP) values were calculated for each predicted sample. The SHAP method, which is derived from game theory, was used to quantify the contribution of each feature in the model toward increasing or decreasing the probability of a single output.

### Statistical analysis

Statistical analyses were conducted using R (version 4.0.2; https://www.r-project.org) and Python (version 3.7.2; https://www.python.org). Continuous variables are presented as mean ± standard deviation, whereas categorical variables are expressed as counts (n) and percentages (%). Categorical data were compared using the chi-square test; normally distributed continuous variables were assessed using the Student t-test, and non-normally distributed continuous variables among multiple groups were analyzed using the Kruskal–Wallis H-test. The model’s predictive performance was assessed by evaluating the AUC of the subject’s operating characteristic curve, as well as accuracy, sensitivity, specificity values, calibration curves, and decision curve analysis (DCA). In addition, Spearman’s correlation analysis was performed to examine the correlation of radiomics characteristics of arterial and portal phase habitats with clinical features. All statistical tests were two-sided, and a significance level of *P* < 0.05 was used.

## Results

### Patient characteristics

We retrospectively collected 344 cases of hepatic cancer resection from two hospitals. Center 1 included 243 patients with a mean age of 56.19 ± 11.17 years. Patients in center 1 were divided into a training set (n = 170) and an internal validation set (n = 73) in a ratio of 7:3 using random assignment. Center 2 included 101 patients with a mean age of 57.64 ± 9.19 years. Patients in center 2 comprised the external validation set (n = 101). The demographic and clinical characteristics of the training set, internal validation set, and external validation set were compared. The results showed that all variables had a *P* value of >0.05, and the difference was not statistically significant. The data were comparable among the three sets, as shown in **Table 1**. The univariable LR analysis identified age, number of tumors, and AFP as clinically relevant risk factors, as shown in **Table 2**.

**Table 1:** Demographics and clinical characteristics.

Feature name		Train (n=170)	Internal validation (n=73)	External validation (n=101)	P value
Tbil, μmol/L		19.67 ± 23.13	18.87 ± 18.60	22.35 ± 27.53	0.195
GGT, U/L		89.59 ± 143.21	112.52 ± 185.12	99.70 ± 160.11	0.923
SII		442.00 ± 596.03	429.29 ± 354.36	369.96 ± 292.91	0.373
LMR		3.55 ± 1.45	3.54 ± 1.35	3.76 ± 1.33	0.077
NLR		2.38 ± 2.09	2.45 ± 2.27	2.17 ± 1.29	0.546
ALR		67.37 ± 54.16	83.94 ± 130.60	67.82 ± 56.87	0.601
PLR		117.63 ± 67.03	125.44 ± 54.96	109.79 ± 48.29	0.144
Age, years					0.900
	<60	102 (60.00)	44 (60.27)	58 (57.43)	
	≥60	68 (40.00)	29 (39.73)	43 (42.57)	
Sex					0.592
	Female	46 (27.06)	17 (23.29)	22 (21.78)	
	Male	124 (72.94)	56 (76.71)	79 (78.22)	
Hypertension					0.967
	No	121 (71.18)	53 (72.60)	73 (72.28)	
	Yes	49 (28.82)	20 (27.40)	28 (27.72)	
Diabetes					0.055
	No	140 (82.35)	69 (94.52)	83 (82.18)	
	Yes	30 (17.65)	4 (5.48)	18 (17.82)	
Cirrhosis					0.081
	No	74 (43.53)	43 (58.90)	46 (45.54)	
	Yes	96 (56.47)	30 (41.10)	55 (54.46)	
Hepatic virus infection					0.773
	No	77 (45.29)	33 (45.21)	50 (49.50)	
	Yes	93 (54.71)	40 (54.79)	51 (50.50)	
Maximum tumor diameter, cm					0.106
	<5	94 (55.29)	26 (35.62)	69 (68.32)	
	≥5	76 (44.71)	47 (64.38)	32 (31.68)	
Number of tumors					0.013
	=1	137 (80.59)	57 (78.08)	94 (93.07)	
	≥2	33 (19.41)	16 (21.92)	7 (6.93)	
AFP, ng/ml					0.908
	<400	110 (64.71)	48 (65.75)	68 (67.33)	
	≥400	60 (35.29)	25 (34.25)	33 (32.67)	
ALB, g/L					0.625
	<40	112 (65.88)	45 (61.64)	61 (60.40)	
	≥40	58 (34.12)	28 (38.36)	40 (39.60)	
PLT, 10^9^/L					0.254
	<125	34 (20.00)	9 (12.33)	22 (21.78)	
	≥125	136 (80.00)	64 (87.67)	79 (78.22)	
AST, U/L					0.463
	<40	112 (65.88)	44 (60.27)	70 (69.31)	
	≥40	58 (34.12)	29 (39.73)	31 (30.69)	
ALT, U/L					0.830
	<50	128 (75.29)	53 (72.60)	73 (72.28)	
	≥50	42 (24.71)	20 (27.40)	28 (27.72)	

**Table 2:** Univariable logistic regression analysis.

feature_name	Log (OR)	Lower 95% CI	Upper 95% CI	OR	OR lower 95% CI	OR upper 95% CI	*p*_value
Age	-0.124	-0.211	-0.037	0.883	0.81	0.964	0.019
Sex	0.025	-0.074	0.125	1.026	0.929	1.133	0.676
Hypertension	-0.003	-0.098	0.093	0.997	0.907	1.097	0.963
Diabetes	0.05	-0.07	0.17	1.051	0.932	1.185	0.494
Cirrhosis	0.091	0.005	0.177	1.095	1.005	1.194	0.08
Hepatic virus infection	0.046	-0.04	0.132	1.047	0.961	1.141	0.38
Maximum tumor diameter	0.085	-0.001	0.171	1.088	0.999	1.186	0.105
Number of tumors	0.142	0.026	0.258	1.153	1.026	1.294	0.044
AFP	0.162	0.073	0.252	1.176	1.076	1.287	0.003
Tbil	-0.001	-0.003	0.001	0.999	0.997	1.001	0.343
ALB	-0.044	-0.133	0.046	0.957	0.875	1.047	0.421
PLT	-0.019	-0.129	0.091	0.981	0.879	1.095	0.777
AST	0.085	-0.006	0.175	1.088	0.994	1.191	0.123
ALT	0.064	-0.034	0.161	1.066	0.967	1.175	0.284
GGT	0	0	0	1	1	1	0.698
SII	0	0	0	1	1	1	0.464
LMR	-0.005	-0.036	0.025	0.995	0.965	1.025	0.773
NLR	0.01	-0.013	0.032	1.01	0.987	1.033	0.48
ALR	0	0	0.001	1	1	1.001	0.501
PLR	0	0	0.001	1	1	1.001	0.574
ALBI	0.021	-0.074	0.117	1.021	0.929	1.124	0.716

### Feature filtering

**Figure 3:**
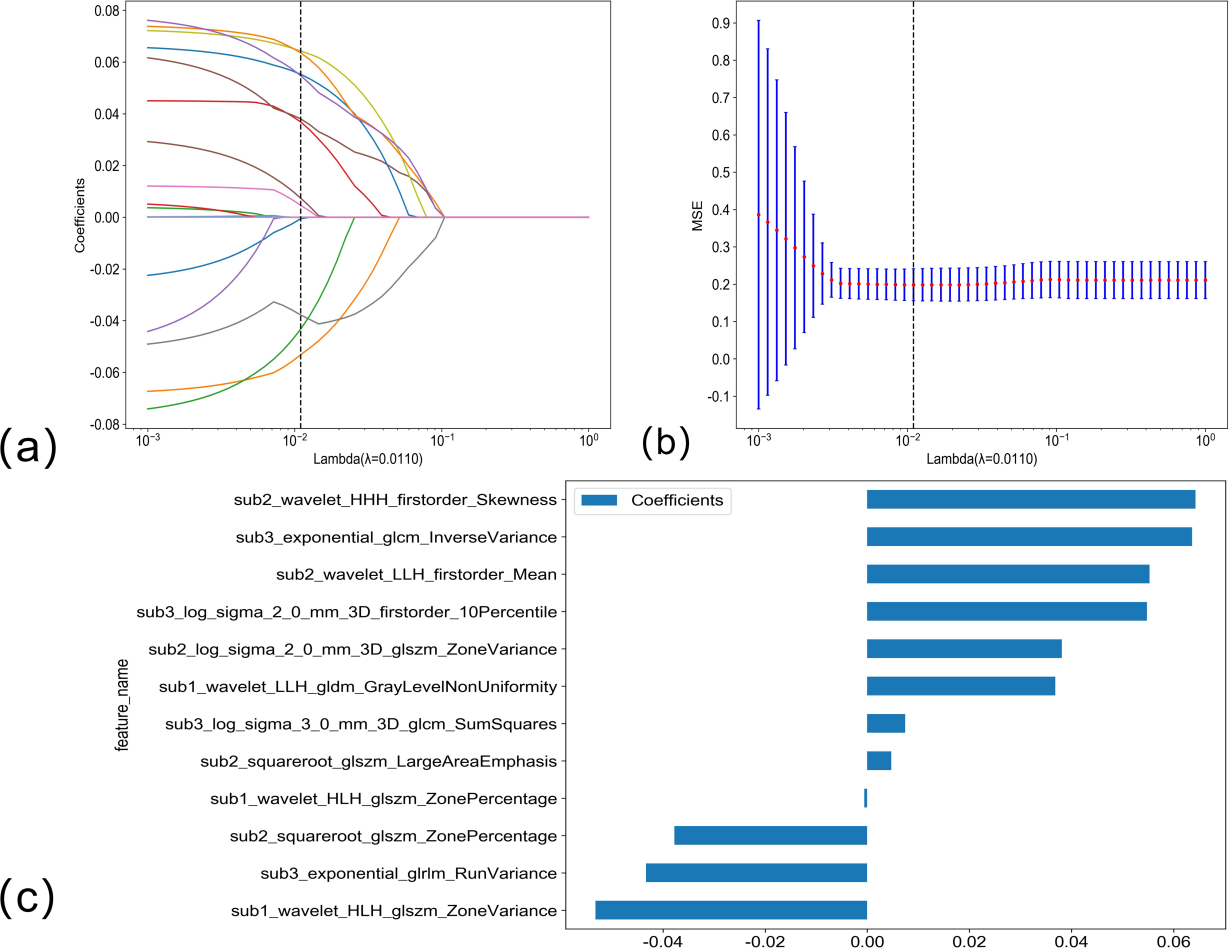
**(A, B)** Arterial phase habitat radiomics feature selection using the LASSO regression. LASSO coefficient profiles of the 12 candidate habitat radiomics features. Optimal λ was identified using 10-fold cross-validation based on the minimum value criterion. **(C)** The histogram of the feature importance: the Y-axis indicates the selected 12 features, and the X-axis represents the coefficient of arterial phase habitat radiomics.

The LASSO regression analysis model was used for dimensionality reduction of arterial phase habitat radiomics features; the selection of penalty coefficients (λ = 0.0110) and the graphs of the feature screening process and feature coefficients with λ are shown in **Figure 3**. After the final screening of arterial phase habitat radiomics features, 12 characteristics were retained.

**Figure 4:**
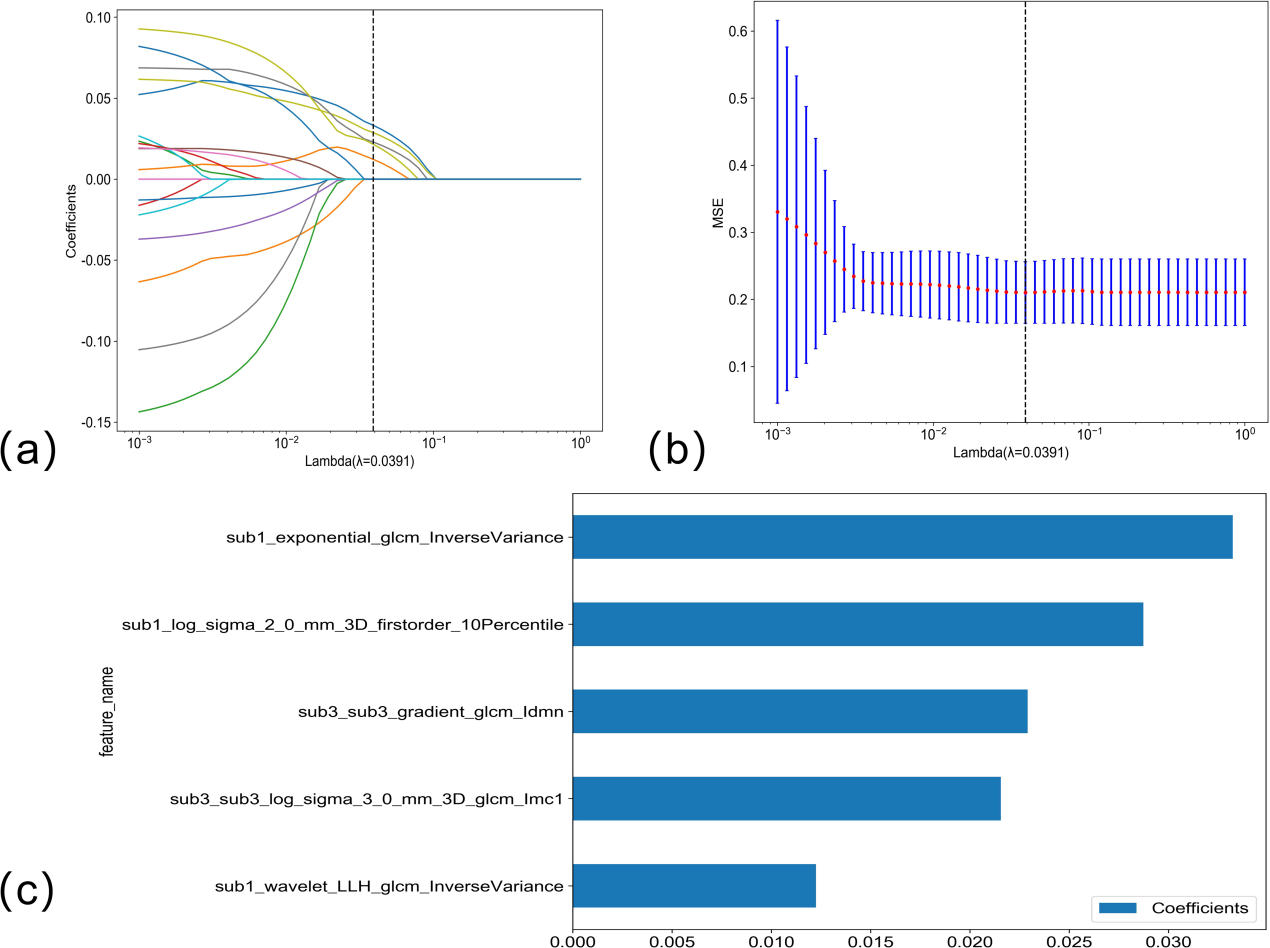
**(A, B)** Venous phase habitat radiomics feature selection using LASSO regression. LASSO coefficient profiles of the five candidate habitat radiomics features. Optimal λ was identified using 10-fold cross-validation based on the minimum value criterion. **(C)** The histogram of the feature importance: the Y-axis indicates the selected five features, and the X-axis represents the coefficient of portal phase habitat radiomics.

The LASSO regression analysis model was used to perform dimensionality reduction on the radiomics features of portal phase habitat; the selection of the penalty coefficient (λ = 0.0391) and the graphs of the feature screening process, as well as the variation of the feature coefficient with λ, are shown in **Figure 4**. After the final screening of the radiomics features of portal phase habitat, five features were retained.

### Model performance and validation

The optimal model among the clinical models was XGBboost, as detailed in Attachment S1. The training set accuracy, sensitivity, specificity, and AUC were 0.529, 0.442, 0.740, and 0.616 (95% CI: 0.5281–0.7041), respectively. The internal validation set accuracy, sensitivity, specificity, and AUC were 0.634, 0.468, 0.618, and 0.639 (95% CI: 0.5338–0.7433), respectively, and the external validation set accuracy, sensitivity, specificity, and AUC were 0.616, 0.500, 0.793, and 0.649 (95% CI: 0.5258–0.7712), respectively.

The optimal model in arterial phase habitat radiomics was MLP, as detailed in Attachment S2. The training set accuracy, sensitivity, specificity, and AUC were 0.741, 0.750, 0.720, and 0.766 (95% CI: 0.6864–0.8466), respectively. The internal validation set accuracy, sensitivity, specificity, and AUC were 0.614, 0.717, 0.500, and 0.569 (95% CI: 0.4557–0.6819), respectively, and the external validation set accuracy, sensitivity, specificity, and AUC were 0.658, 0795, 0.448, and 0.615 (95% CI: 0.4832–0.7472), respectively.

The optimal model in portal phase habitat radiomics was LR, as detailed in Attachment S3. The training set accuracy, sensitivity, specificity, and AUC were 0.618, 0.567, 0.740, and 0.688 (95% CI: 0.5991–0.7759), respectively. The internal validation set accuracy, sensitivity, specificity, and AUC were 0.545, 0.321, 0.792, and 0.509 (95% CI: 0.3954–0.6234), respectively, and the external validation set accuracy, sensitivity, specificity, and AUC were 0.699, 0.727, 0.655, and 0.665 (95% CI: 0.5299–0.8009), respectively.

The arterial phase radiomics signature (artery_rad_hab_sign), portal phase radiomics signature (vein_rad_hab_sign), and clinical features were combined to establish the optimal model as SVM, as detailed in Attachment S4. The training set accuracy, sensitivity, specificity, and AUC were 0.876, 0.904, 0.818, and 0.902 (95% CI: 0.8470–0.9579), respectively. The internal validation set accuracy, sensitivity, specificity, and AUC were 0.792, 0.925, 0.646, and 0.817 (95% CI: 0.7335–0.9009), respectively, and the external validation set accuracy, sensitivity, specificity, and AUC were 0.877, 0.898, 0.833, and 0.896 (95% CI: 0.8108–0.9817), respectively.

**Figure 5:**
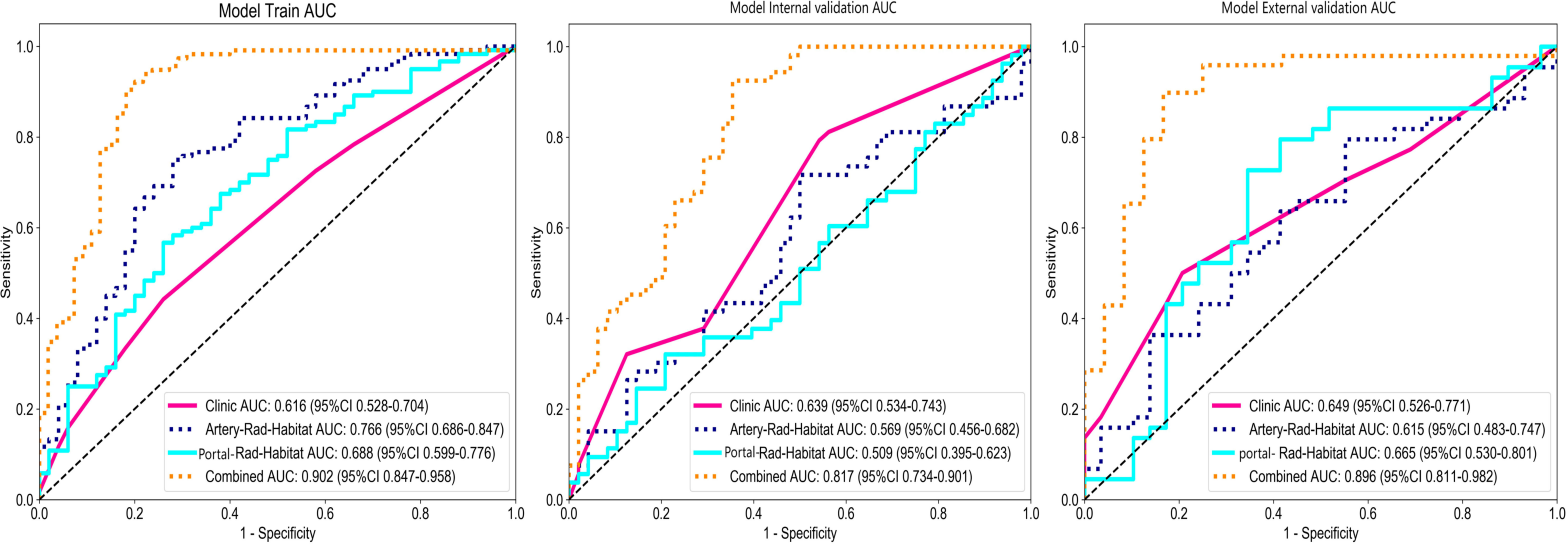
Model ROC curves. The combined model’s AUC in the training set, internal validation set, and external validation set is higher than that of the other models.

**Figure 6:**
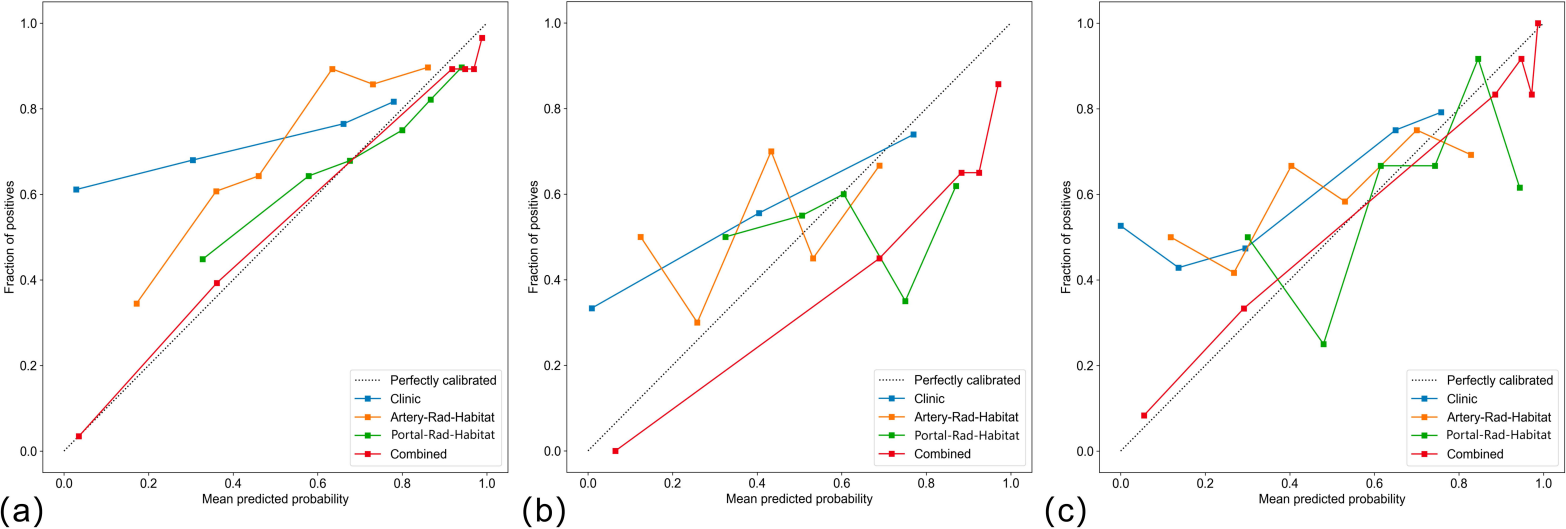
Calibration curves **(A)** training set, **(B)** internal validation, **(C)** external validation only. The combined model predictions are in good agreement with the actual results.

**Figure 7:**
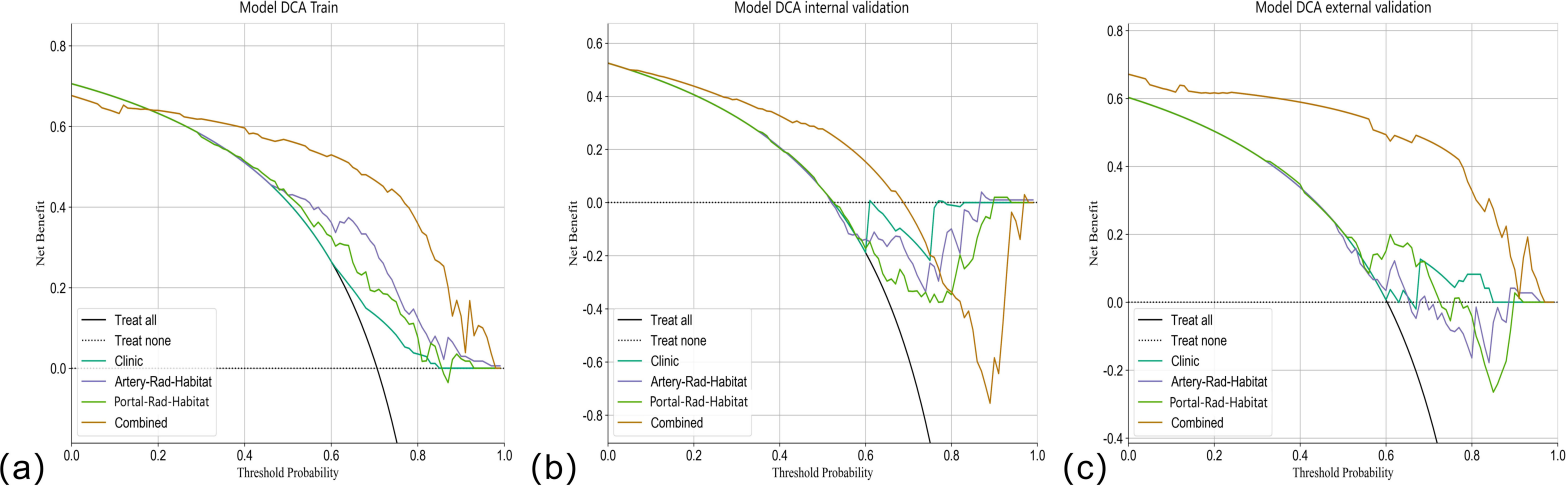
DCA curves. The Y-axis represents the net benefit, and the X-axis shows the threshold probability that the expected benefit of the treatment is equal to the expected benefit of not receiving treatment. In the combined model of the **(A)** training set, **(B)** internal validation set, and **(C)** external validation set, most patients with threshold probability have net benefit.

**Figure 8:**
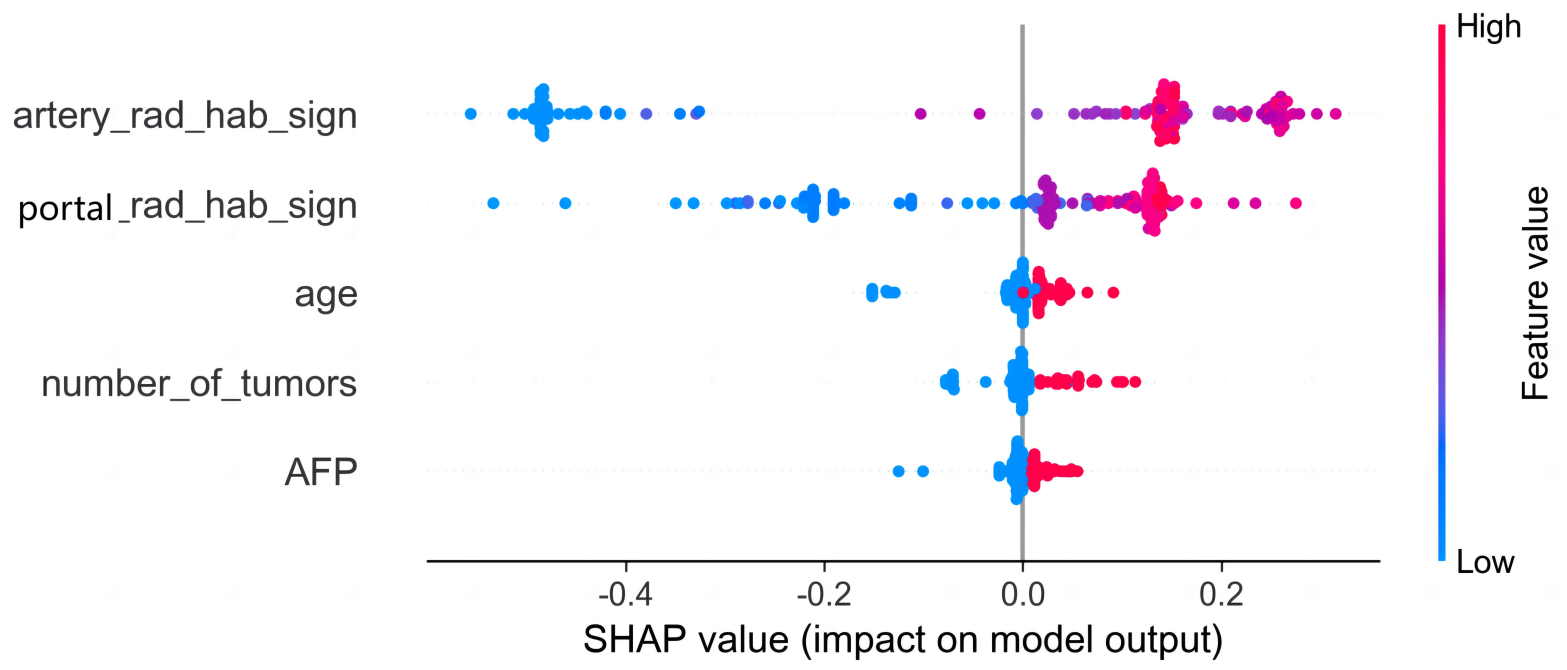
SHAP analysis of the combined model. The color represents the value of the variable, with red representing the larger value and blue representing the smaller value. Arterial phase habitat radiomics signature was the most important risk factor in the combined model.

Among the above models, the combined model emerged as the optimal choice because of its superior accuracy, sensitivity, specificity, and AUC compared to other models (**Table 3**, **Figure 5**). The calibration curves show that only the combined model simultaneously in the training set, internal validation set, and external validation set predicted results in better agreement with the actual results (**Figure 6**). In addition, the DCA comparing the training set, internal validation set, and external validation set showed that only the combined model had a net gain of patients at most threshold probabilities (**Figure 7**). According to the SHAP value analysis, artery-rad-hab-sign was the most important risk factor in the combined model (**Figure 8**).

**Table 3:** Model performance comparison.

model_name	Accuracy	AUC (95% CI)	Sensitivity	Specificity	Task
Clinic	0.529	0.616 (95% CI: 0.5281-0.7041)	0.442	0.740	Train
Clinic	0.634	0.639 (95% CI: 0.5338-0.7433)	0.468	0.618	Internal validation
Clinic	0.616	0.649 (95% CI: 0.5258-0.7712)	0.5000	0.793	External validation
Artery-Rad-Habitat	0.741	0.766 (95% CI: 0.6864-0.8466)	0.750	0.720	Train
Artery-Rad-Habitat	0.614	0.569 (95% CI: 0.4557-0.6819)	0.717	0.500	Internal validation
Artery-Rad-Habitat	0.658	0.615 (95% CI: 0.4832-0.7472)	0795	0.448	External validation
Portal-Rad-Habitat	0.618	0.688 (95% CI: 0.5991-0.7759)	0.567	0.740	Train
Portal-Rad-Habitat	0.545	0.509 (95% CI: 0.3954-0.6234)	0.321	0.792	Internal validation
Portal-Rad-Habitat	0.699	0.665 (95% CI: 0.5299-0.8009)	0.442	0.740	External validation
Combined	0.876	0.902 (95% CI: 0.8470-0.9579)	0.904	0.818	Train
Combined	0.792	0.817 (95% CI: 0.7335-0.9009)	0.925	0.646	Internal validation
Combined	0.877	0.896 (95% CI: 0.8108-0.9817)	0.898	0.833	External validation

### Correlation of radiomics characteristics with clinical factors

The results of the correlation study, which examined the clinical features in relation to the characteristics of the arterial and portal phases, showed that during the arterial phase, age showed a significant negative correlation with sub3_log_sigma_2_0_mm_3D_firstorder_10Percentile and sub2_wavelet_LLH_firstorder_Mean (**Supplementary Figure S1**). Moreover, MVI showed a significant positive correlation with sub3_exponential glrlm_RunVariance, sub2_wavelet_HHH_firstorder_Skewness, and sub1_wavelet_LLH_gldm_GrayLevelNonUniformity. In the portal phase, APF and MVI were positively correlated with sub3_sub3 gradient_glcm_Idmn and sub3_sub3 Log-sigM-2-0-MM3D_GLcm_IMC1. There was a significant negative correlation between age and sub1_log-sigma-2-0-mm-3D_firstonder_10Percentile. In the portal phase, APF and MVI were positively correlated with sub3_sub3 gradient_glcm_Idmn and sub3_sub3 log-sigm-2-0-mm-3D_glcm_Imc1. There was a significant negative correlation between age and sub1_log-sigma-2-0-mm-3D_firstonder_10Percentile.

## Discussion

The high incidence of postoperative recurrence of HCC poses a significant challenge to patient prognosis. Early recurrence is among the most crucial factors impacting the prognosis of HCC (18). Preoperative adjuvant therapy may mitigate the risk of HCC recurrence; however, clear clinical models and methods for selecting potential candidates are currently lacking. Furthermore, liver transplantation remains the primary treatment option for liver cancer. By identifying and evaluating patients at high risk for postoperative recurrence, we can optimize the allocation of limited organ resources and enhance their long-term prognosis (19). A study conducted by Gu et al. (20) revealed that up to 30% of patients with a high risk of postoperative recurrence developed distant metastases, whereas the recurrence rate for extrahepatic tumors was as high as 97.7%. Consequently, implementing screening measures for individuals at a heightened risk of early postoperative HCC recurrence can facilitate the development of personalized treatment strategies and postoperative surveillance programs, ultimately enhancing patient survival.

In recent years, several studies have focused on early postoperative HCC recurrence on the basis of CT images, investigating the correlation between HCC recurrence and radiomics features with promising predictive outcomes (21–23). However, most studies have solely focused on extracting radiomics features from the entire tumor region. This approach may introduce confounding factors like hemorrhage, necrosis, cystic changes, and edema, which can confound the heterogeneous expression and subsequently compromise the predictive accuracy of the model. The emerging technique of habitat analysis in imaging focuses on subregional histology analysis, enabling more precise quantification of tumor subregions associated with growth or invasiveness (24). This method reveals the spatial heterogeneity of tumors, distinguishing it from overall tumor radiomics analysis.

In the present study, to objectively extract the internal subregions of the tumor, we used feature extraction using the GLCM for each pixel within the tumor region. Subsequently, to generate three distinct subregions, clustering was performed on these pixels based on their local features. The GLCM effectively captures subtle texture variations in response to irregularities and complexities of the image, which is significant for investigating tumor heterogeneity (15). The division of arterial and portal venous phases was incorporated into the subregion clustering, taking into account the distinct characteristics of tumors during different blood supply phases. This approach ensures accurate habitat mapping by avoiding the omission of specific imaging voxels with varying blood supply phase characteristics due to different sequencing methods. The findings suggest that distinct subregions within the arterial and portal venous phases of enhanced CT images have a certain predictive value for early postoperative HCC recurrence. The AUC values in the training set for arterial and portal venous phases were 0.766 (95% CI: 0.6864–0.8466) and 0.688 (95% CI: 0.5991–0.7759), respectively, indicating a favorable prediction compared to the model based solely on clinical features, which had an AUC value of 0.615 (95% CI: 0.5281–0.7041). Subsequently, we developed a combined model incorporating subregion features from different sequences along with clinical features, which significantly improved prediction performance when compared to individual models alone. The combined model showed superior predictive ability in the training set, internal validation set, and external validation set, with AUC values of 0.902 (95% CI: 0.8470–0.9579), 0.817 (95% CI: 0.7335–0.9009), and 0.896 (95% CI: 0.8108-0.9817), respectively, highlighting their complementary nature in enhancing personalized diagnosis. In addition, the calibration curves of the combined model showed remarkable consistency between the predicted and actual results. Moreover, the DCA curves showed that the combined model holds substantial clinical significance in predicting early recurrence of HCC. These findings suggest that our study offers valuable insights for HCC management.

The SHAP method was used to analyze the individual contribution of each feature in the model toward the final prediction of the observation (25). This analysis significantly enhanced the interpretability of the machine learning model (26). The findings revealed that arterial phase habitat radiomics features were the most influential risk factors, followed by portal phase radiomics features. This indicates that hemodynamics and microenvironment of HCC are associated with aggressive biological behavior (27) and that early recurrence may be attributed to tumor aggressiveness. The analysis of the correlation between the arterial and portal phase habitat radiomics and the clinical features revealed a close association of different subregional areas in each phase with age, AFP, and MVI (**Supplementary Figure S1**). This suggests varying levels of invasiveness among different subregions. Furthermore, it is notable that most of our extracted features were derived from wavelet features, which effectively capture heterogeneity at multiple spatial scales (28, 29).

The heterogeneity within a tumor, which can be observed as local or global differences on CT images, is likely attributed to variances in tumor cell composition or properties. These habitat analysis features offer more precise information on the aggressive characteristics of HCC and enable better quantification of tumor subregions closely associated with growth or aggressiveness (24). This further highlights the significance of using habitat analysis techniques to characterize the intricate microenvironment of tumors. Invasive subregions reportedly play a crucial role in prognosis and treatment response medicine.

This study used a habitat analysis approach to integrate radiomics features from diverse tumor subregions with clinical variables, resulting in better predictive performance than models solely using radiomics features or clinical data. The study was conducted across multiple centers, and the model’s predictive performance was validated by testing it on an external dataset, demonstrating its robustness across different datasets and scanners. However, this study has several limitations. This was a retrospective study lacking precise correspondence between CT image habitats and pathology samples, necessitating more comprehensive prospective studies to investigate the behavior of biology within each habitat. The definition of manual segmentation boundaries can be contentious because of subjective observer tendencies when outlining tumors. Furthermore, the current study did not explore the correlation between genomic profiles and radiomic phenotypes; thus, future research should incorporate additional dimensions of data to further enhance the model’s predictive performance.

In conclusion, habitat analysis enables the quantification and visualization of distinct subregions within the tumor, providing valuable information for predicting early postoperative recurrence of HCC. We herein successfully developed a combined model that can preoperatively predict early postoperative recurrence of HCC on the basis of subregional features of CT images and clinical characteristics. The external validation set confirms the model’s stable and strong predictive performance. This can provide a more accurate basis for the development of clinical treatments and monitoring programs.

## Data Availability

The original contributions presented in the study are included in the article/**Supplementary Material**. Further inquiries can be directed to the corresponding author.

## Supplementary material

Supplementary Figure 1 A heatmap of the correlation between arterial and portal phase radiomics features and clinical factors. **(A)** A correlation heatmap of arterial phase habitat radiomics features with clinical factors; **(B)** a correlation heatmap of portal phase habitat radiomics features with clinical factors and correlations.

## Glossary

HCC: hepatocellular carcinoma
CT: computed tomography
RFA: radiofrequency ablation
TACE: transarterial chemoembolization
Tbil: total bilirubin
GGT: γ-glutamyl transferase, SII, systemic immune-inflammation index
LMR: lymphocyte-to-monocyte ratio
NLR: neutrophil-to-lymphocyte ratio
ALR: aspartate aminotransferase-to-lymphocyte ratio
PLR: platelet to lymphocyte ratio
AFP: alpha-fetoprotein
ALB: albumin
PLT: platelet count
AST: aspartate transaminase
ALT: alanine aminotransferase
TTR: the time to recurrence
GLCM: Gray-Level Co-occurrence Matrix
LASSO: least absolute shrinkage and selection operator
LR: logistic regression
SVM: support vector machine
DT: decision tree
RF: random forest
XGBoost: extreme gradient boosting
MLP: multilayer perceptual machine
SHAP: SHapley Additive exPlanations
DCA: decision curve analysis
MVI: Microvascular cancer thrombus
ROC: receiver operating characteristic
