# Supplementary figures and images for: Prediction of early postoperative recurrence of hepatocellular carcinoma by habitat analysis based on different sequence of contrast-enhanced CT
Source: Front Oncol. 2025 Jan 3;14:1522501. doi: 10.3389/fonc.2024.1522501 (PMC11739309; doi:10.3389/fonc.2024.1522501)

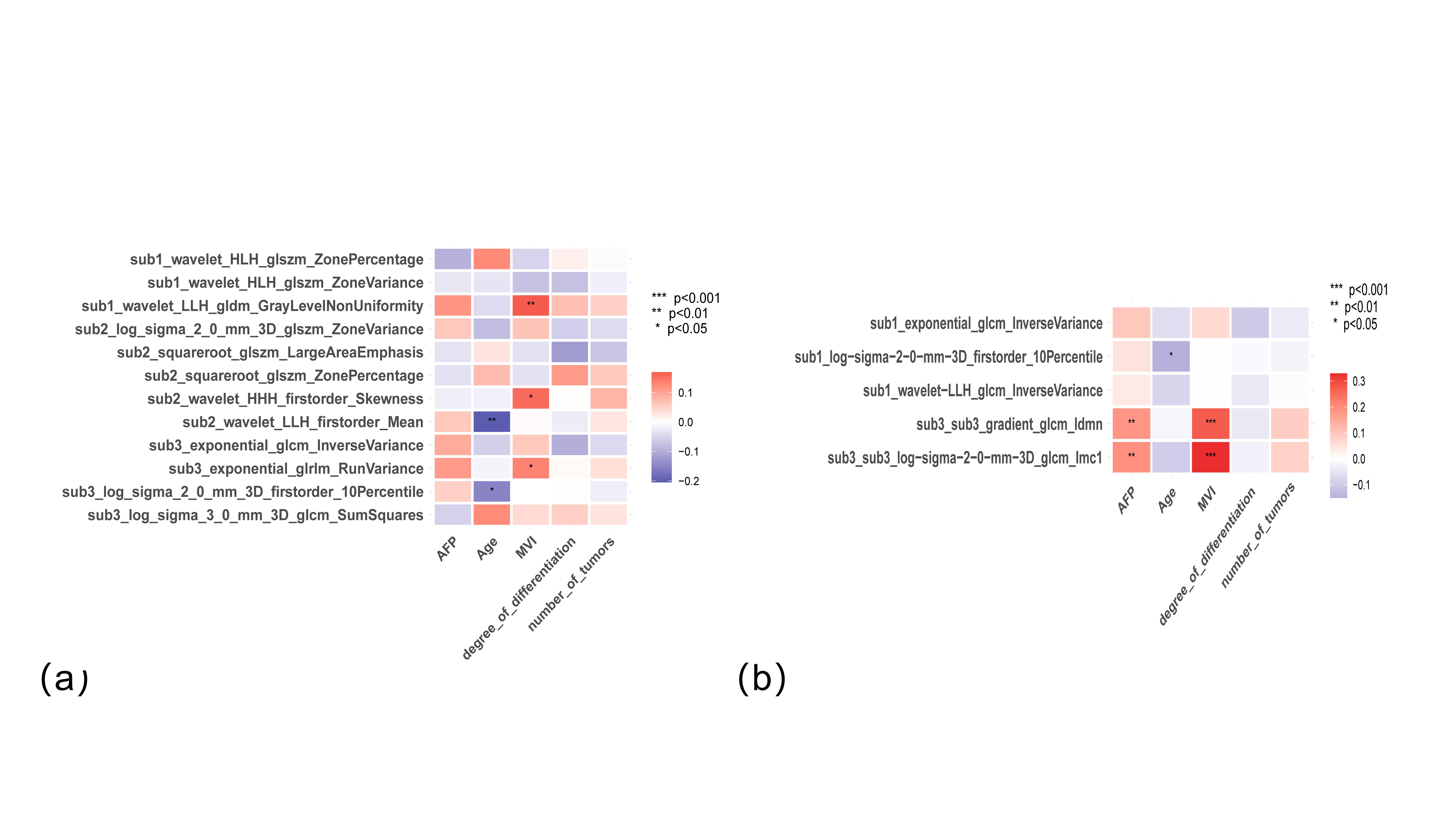

Supplement: Supplementary Figure 1 — A heatmap of the correlation between arterial and portal phase radiomics features and clinical factors. (A) A correlation heatmap of arterial phase habitat radiomics features with clinical factors; (B) a correlation heatmap of portal phase habitat radiomics features with clinical factors and correlations. [file Image1.jpeg]
